# Effective specialist or jack of all trades? Experimental evolution of a crop pest in fluctuating and stable environments

**DOI:** 10.1111/eva.13360

**Published:** 2022-03-08

**Authors:** Anna Skoracka, Alicja Laska, Jacek Radwan, Mateusz Konczal, Mariusz Lewandowski, Ewa Puchalska, Kamila Karpicka‐Ignatowska, Anna Przychodzka, Jarosław Raubic, Lechosław Kuczyński

**Affiliations:** ^1^ 49562 Population Ecology Lab Faculty of Biology Institute of Environmental Biology Adam Mickiewicz University Poznań Poland; ^2^ 49562 Center for Advanced Technology Adam Mickiewicz University Poznań Poland; ^3^ 49562 Evolutionary Biology Group Faculty of Biology Institute of Environmental Biology Adam Mickiewicz University Poznań Poland; ^4^ Section of Applied Entomology Department of Plant Protection Institute of Horticultural Sciences Warsaw University of Life Sciences – SGGW Warsaw Poland

**Keywords:** *Aceria tosichella*, environmental variability, experimental evolution, host range, niche breadth, wheat curl mite

## Abstract

Understanding pest evolution in agricultural systems is crucial for developing effective and innovative pest control strategies. Types of cultivation, such as crop monocultures versus polycultures or crop rotation, may act as a selective pressure on pests’ capability to exploit the host’s resources. In this study, we examined the herbivorous mite *Aceria tosichella* (commonly known as wheat curl mite), a widespread wheat pest, to understand how fluctuating versus stable environments influence its niche breadth and ability to utilize different host plant species. We subjected a wheat‐bred mite population to replicated experimental evolution in a single‐host environment (either wheat or barley), or in an alternation between these two plant species every three mite generations. Next, we tested the fitness of these evolving populations on wheat, barley, and on two other plant species not encountered during experimental evolution, namely rye and smooth brome. Our results revealed that the niche breadth of *A. tosichella* evolved in response to the level of environmental variability. The fluctuating environment expanded the niche breadth by increasing the mite’s ability to utilize different plant species, including novel ones. Such an environment may thus promote flexible host‐use generalist phenotypes. However, the niche expansion resulted in some costs expressed as reduced performances on both wheat and barley as compared to specialists. Stable host environments led to specialized phenotypes. The population that evolved in a constant environment consisting of barley increased its fitness on barley without the cost of utilizing wheat. However, the population evolving on wheat did not significantly increase its fitness on wheat, but decreased its performance on barley. Altogether, our results indicated that, depending on the degree of environmental heterogeneity, agricultural systems create different conditions that influence pests’ niche breadth evolution, which may in turn affect the ability of pests to persist in such systems.

## 1 INTRODUCTION

Agricultural development and expansion have been two of the most important steps in the development of civilization and one of the major drivers changing the functioning of many ecosystems. The domestication of many plant species and selective breeding, aimed at increasing yields, has led to remarkable boosts in food production, which has supported the growing human population (Foley et al., 2005; Khush, 2001). However, agriculture also has broader evolutionary consequences because it drives evolutionary change in not only domesticated plants but also other species associated with croplands, such as plant pests. Various agricultural practices may create different selective environments that influence pests’ life‐history traits and tolerance to biotic and abiotic factors, and thus, affect their harmfulness and invasiveness (Palumbi, 2001).

Over a single season, due to different agricultural practices, herbivores may face homogeneous environments in the form of monoculture crops (involving only a particular crop at a time in a specific field) or heterogeneous environments in the form of polycultures (involving two or more crops at a time). Environmental heterogeneity may also be created over a longer time scale by temporal (e.g., annual) crop rotation, that is, by growing different crops sequentially. Such contrasting environmental conditions of “stability versus variability” may affect the evolution of the niche breadth of pests. Intuitively, niche breadth should evolve to match the amount of environmental variation (Futuyma & Moreno, 1988; Maynard Smith & Hoekstra, 1980; Scheiner, 1993; Via & Lande, 1985). Therefore, homogeneous environments, such as monocultures, should favor specialization, that is, exploitation of a restricted set of resources. In contrast, heterogeneous environments, such as those created by crop rotation, should select for the ability to use multiple resources, that is, generalization (e.g., Felsenstein, 1976; Futuyma & Moreno, 1988; Hardy et al., 2020; Kassen, 2002; Levins, 1968). A generalist species can either refer to a diverse collection of specialized phenotypes, which reflects the evolutionary history of adaptation to a wide range of specific resources, or a population of open‐ended phenotypes capable of managing stress induced by a broad range of environments (Bolnick et al., 2003; Devictor et al., 2010; Hardy et al., 2020; Roughgarden, 1972). In the latter case, generalists may be particularly capable of invading novel hosts to which they have not had a chance to adapt before (Hardy, 2017; Nylin & Janz, 2009).

The expectation that broad niches evolve in spatially or temporally heterogeneous environments, whereas narrow niches evolve in homogeneous environments has been supported by some empirical tests (e.g., Bennett et al., 1992; Condon et al., 2014; Kassen & Bell, 1998; Ketola et al., 2013; Legros & Koella, 2010; Reboud & Bell, 1997; Sant et al., 2021; Venail et al., 2011). However, most studies have not provided a clear evidence of this phenomenon (e.g., Barrett et al., 2005; Bell, 1997; Crill et al., 2000; Ehiobou & Goddard, 1989; Joshi & Thompson, 1997; Reboud & Bell, 1997; Riddle et al., 1986; Scheiner & Yampolsky, 1998). These ambiguous results most likely resulted because the discrepancy between the spatial and temporal scales of environmental variation, and the time when an individual encounters the varying environments during its lifetime can produce divergent evolutionary outcomes (Gomulkiewicz & Kirkpatrick, 1992; Kassen, 2002; Sexton et al., 2017). For example, according to classic models, temporal heterogeneity is expected to select generalists more readily than spatial heterogeneity because phenotypes are obligated to experience each resource one by one over time. In contrast, spatial variation should maintain specialization because, under such conditions, spatially distributed environments may provide refuges for more specialized phenotypes, thus, weakening the selection on wider niche breadths (Kassen, 2002; Levins, 1962, 1965, 1968; Lynch & Gabriel, 1987). A similar outcome is expected when spatial variation is accompanied with low temporal variation, or the absence thereof. Spatially variable environments, however, can also select for wider niches when there is a sufficient rate of dispersal between resource patches, or when the temporal variation within a generation is high (Lynch & Gabriel, 1987; Sultan & Spencer, 2002).

The knowledge of whether an organism is adapted to a restricted spectrum, or rather a wide range of resources, is of crucial importance for understanding its role in the ecosystem. Notably, agricultural systems are generally more homogeneous than the natural habitats they have replaced; still, some practices increase their heterogeneity in space (polycultures, set‐asides) or time (crop rotation). Therefore, it is important to determine whether and how homogeneous and heterogeneous conditions influence the niche breadth of agricultural pests (in terms of host specialization), thereby allowing them to utilize either a narrow subset or a broad range of potential crop resources.

The evolution of these two strategies, namely specialization and generalization, can be driven by the trade‐off between the ability to exploit individual hosts optimally and the ability to utilize the maximum number of hosts (Futuyma & Moreno, 1988; Jaenike, 1990; Kassen, 2002; Via, 1990). Generally, specialists gain the ability to detect hosts quickly, overcome plant defense mechanisms, and manipulate hosts for their benefit. However, this may come at the cost of a narrow niche (restricted range of hosts) (Ali & Agrawal, 2012; Fry, 1990; Petanović & Kielkiewicz, 2010; Whittaker & Feeny, 1971). Instead, generalists gain access to a wide range of host resources, which minimizes the risk of not finding a host and thus the risk of extinction (Ali & Agrawal, 2012; Barrett & Heil, 2012; Bernays & Minkenberg, 1997; Hardy et al., 2020). However, this may also entail costs, in terms of efficiency in utilizing particular hosts (expressed by the “jack of all trades is a master of none” principle) (Futuyma & Moreno, 1988; Joshi & Thompson, 1995; Kawecki & Ebert, 2004; MacArthur, 1972; MacArthur & Pianka, 1966). Notably, the costs incurred by specialists and generalists may be due to limitations in their physiology, morphology, or development (Poisot et al., 2011). The most commonly considered mechanism for the trade‐offs and costs of adaptation stems from antagonistic pleiotropy: alleles that have a beneficial effect in one environment concomitantly have a deleterious effect in other environments (Fry, 1996; Kassen, 2002; Kawecki & Ebert, 2004; Ravigné et al., 2009). These theoretical assumptions, however, have been questioned (Joshi & Thompson, 1995; Remold, 2012). Furthermore, the antagonistic pleiotropy hypothesis received limited empirical support. Few studies have shown that the genetic correlation in performance between different resources is negative (Cooper & Lenski, 2000; Legros & Koella, 2010; MacLean et al., 2004; Via, 1991). However, the majority of research has suggested weak or no trade‐offs in resource exploitation, or even a positive correlation in fitness between different resources (e.g., Agosta & Klemens, 2009; Bedhomme et al., 2012; Caballero et al., 2001; Draghi, 2021; Fox & Caldwell, 1994; Friberg & Wiklund, 2009; Fry, 2001; Futuyma & Philippi, 1987; Gompert et al., 2015; Hereford, 2009; Huey & Hertz, 1984; Magalhães et al., 2009; Vorburger, 2005). This suggests that trade‐offs may not always be the primary cause of specialization. In addition to trade‐offs, demographic events and population‐level processes have been proposed to influence the evolution of niche breadth. This includes, for example, insufficient genetic variation for selection toward optimal genotypes, bottlenecks, genetic drift, assortative mating, habitat choice, or density‐dependent competition (Futuyma et al., 1995; Hardy et al., 2016, 2020; Ravigné et al., 2009; Sargent & Otto, 2006; Sexton et al., 2017; Whitlock, 1996).

Given the plethora of mechanisms that may drive niche breadth evolution, determining how the variability of agricultural systems may affect the niche breadth of pests is not easy. However, understanding how agricultural practices could affect niche breadth evolution, and thereby the potential to exploit crop plant resources, has important practical applications. Specialized herbivores are more likely to find and remain in pure crop stands that ensure concentrated resources and monotonous conditions and are generally more abundant in monocultures than polycultures (Altieri, 1999; Andow, 1991). However, after harvest, when the main host is unavailable for several months, the potential of having a greater tolerance to utilize several resources proves to be useful. This may explain why generalists, who are able to utilize different resources, are abundant in both monocultures and polycultures. In fact, generalists dominate the fauna of agricultural pests (Kennedy & Storer, 2000; Stamps & Linit, 1998). This raises the question of whether pests associated with agriculture gain more from being generalists, or is it contingent on agricultural practices creating homogeneous versus heterogeneous (spatially or temporally) agricultural environmental conditions? Is there a cost of specialization and/or generalization, and how does it affect pest population dynamics (and thus, invasive potential)?

In this study, we address some of these important questions using a species of global economic importance, *Aceria tosichella* Keifer (wheat curl mite). We investigated whether temporally stable or variable environments (plant species) drive the evolution of specialization versus generalization, and whether there are trade‐offs involved in the evolution of niche breadth. The species analyzed in our study was primarily associated with *Triticum aestivum* L. (wheat). Because *T. aestivum* mainly grows in monocultures, after its harvest at maturity pests are forced to find other resources. Therefore, *A*. *tosichella* populations are exposed to homogeneous environmental conditions over a short time scale and heterogeneous environmental conditions over a longer time scale. Here, we reported on the experimental evolution of mite populations on one plant species (corresponding to a constant environment) and two alternating plant species (corresponding to a fluctuating environment). The ancestral population (stock) originated from individuals sampled from wheat; thus, the stock was potentially preadapted to wheat. We exposed the stock to wheat or *Hordeum vulgare* L. (barley) or the alternation between these two hosts. Using this approach, we tested the following hypotheses:

H1 1 Environmental fluctuations in agricultural systems lead to the widening of ecological niches by selection for a broad host range (generalization). In this case, the population evolving in the alternating environment should have higher fitness on barley compared to the ancestral population (Table 1, prediction H1.a), and on wheat it should have similar fitness to the ancestral population (Table 1, prediction H1.b).

H2 2 Evolution toward generalization leads to niche expansion, which includes the ability to utilize plant species that were not encountered previously (unfamiliar hosts). Thus, we expect that the population evolving in the alternating environment will have higher fitness on unfamiliar hosts than the populations evolving in constant environments on those hosts (Table 1, H2.a. 1–4).

H3 3 Herbivores capable of utilizing several hosts pay the costs of generalization by being less fit than the specialists on those hosts. Therefore, the population evolving in the alternating environment should have lower fitness on wheat than the population evolving on wheat (Table 1, H3.a) and lower fitness on barley than the population evolving on barley (Table 1, H3.b).

H4 4 A constant host environment selects for increased efficiency of utilizing this environment. In this case, we expect that the population evolving on barley will have higher fitness on barley than the ancestral population (Table 1, H4.a), and the population evolving on wheat will have higher fitness on wheat than the ancestral population (Table 1, H4.b). However, it must be noted that the ancestral population was established from the initial populations collected from wheat, and it was maintained on wheat before the experimental evolution; therefore, the ancestral population could have reached its limit with respect to its adaption to wheat.

H5 5 Adaptation to a single host leads to the narrowing of niche breadth, and specialists pay the costs of specialization, expressed as a reduced performance on the other plant species. Therefore, we predict that on wheat, the population evolving on barley will exhibit lower fitness than the population evolving in the alternating environment (Table 1, H5.a). Correspondingly, on barley, the population evolving on wheat will have lower fitness than the population evolving in an alternating environment (Table 1, H5.b).

**TABLE 1:** Contrasts reflecting predictions with expected direction (equal to zero, above zero, or below zero)

Hypothesis	Prediction	Contrast	Direction
**H1**	aTH should have a broader niche than stock, i.e., should have higher fitness on H compared to stock aTH should maintain ability to utilize T	H aTH − H STOCK T aTH − T STOCK	>0
			=0
**H2**	aTH should have higher fitness on unfamiliar hosts (B and S) compared to fitness of cH and cT on these hosts	B aTH − B cH B aTH − B cT S aTH − S cH S aTH − S cT	>0
**H3**	aTH should have lower fitness on H compared to cH aTH should have lower fitness on T compared to cT	H aTH − H cH T aTH − T cT	<0
			<0
**H4**	cH should have higher fitness on H compared to stock cT should have higher fitness on T compared to stock	H cH − H STOCK T cT − T STOCK	>0
			>0
**H5**	cH should have lower fitness on T compared to aTH cT should have lower fitness on H compared to aTH	T cH − T aTH H cT − H aTH	<0
			<0

Note

Explanation of the codes: Plant species: T, *Triticum aestivum* (wheat); H, *Hordeum vulgare* (barley); B, *Bromus inermis* (brome); S, *Secale cereale* (rye). Populations: stock, ancestral population collected from wheat; aTH, population evolving on alternating T and H; cH, population evolving on H; cT, population evolving on T. Contrasts are differences in population growth rates (*r*) for respective combinations of plant species and experimental populations. For example, “H aTH − H cH” is the difference in *r* estimated on *Hordeum* in the population that evolved in an alternating regime versus *r* estimated on *Hordeum* in the population evolved in a constant regime on *Hordeum*.

We found that the fluctuating environment broadened the niche breadth of a specialist crop pest by increasing its fitness on different plant species, including novel ones; however, this phenomenon was associated with some costs. The constant environment enhanced the ability to exploit the particular plant resources effectively. Thus, our experiment contributes to understanding the mechanisms that influence a pest’s ability to persist and thrive in agricultural environments, characterized by both stable and fluctuating conditions.

## 2 MATERIALS AND METHODS

### 2.1 Experimental system

We performed an evolutionary experiment on a wheat‐bred population of *Aceria tosichella* (wheat curl mite). It is one of the most important pests found in cereals and the primary vector of *Wheat streak mosaic virus* (WSMV) and several other plant viruses. Its small size enables the species to be undetected when infesting commodities; additionally, it has the ability to spread over long distances in wind currents and possesses broad thermal tolerance, which improves the mite’s invasive potential (Kuczyński et al., 2016; Navia et al., 2013). The mite represents a useful model species in ecology and evolution because it favors easy laboratory manipulation and maintenance of populations, has a relatively short generation time (7–10 days in 22–27ºC), rapid population growth, amenability to experimental evolution, and responds well to standard laboratory rearing protocols (e.g., Karpicka‐Ignatowska et al., 2019, 2021; Kuczyński et al., 2016; Laska et al., 2021).

*Aceria tosichella* has long been considered a host generalist, infesting approximately 100 species of grasses (Navia et al., 2013). However, DNA barcoding has revealed that the mite represents a complex of cryptic species consisting of at least 29 genetically divergent lineages with differing ecological traits, such as host plant range, thermal optima, and the ability to transmit viruses (Kuczyński et al., 2016; McMechan et al., 2014; Skoracka, Lopes, et al., 2018; Skoracka et al., 2018). In this study, we conducted experiments on the MT‐1 genotype, known as type 2 in Australia and North America (Carew et al., 2009; Hein et al., 2012), which is one of the most pestiferous and widespread genotypes that commonly occur in major agricultural areas that cultivate cereals (North and South America, Africa, Europe, Asia, and Oceania) (Navia et al., 2013; Skoracka et al., 2014).

We used MT‐1 individuals from a genetically diverse ancestral (stock) population established in November 2017. To create the stock population, we extensively sampled cereal fields across an area of thousands of km^2^, focusing on sample localities where *A. tosichella* MT‐1 was found in previous years. We visited 85 locations and collected samples of *T*. *aestivum* (wheat), *H*. *vulgare* (barley), *Triticosecale* Wittm. ex A. Camus. (triticale), and *Bromus inermis* Leyss. (smooth brome), each consisting of at least 10 shoots. In the laboratory, we examined the plants under stereomicroscopes and searched for *A. tosichella* individuals, which were found in the samples obtained from 24 locations. From each infested plant (mostly spikes were inhabited by mites), we selected several random individuals and barcoded them (single specimens) using the cytochrome oxidase subunit I (COI) to confirm their MT‐1 genotype. We adopted the Chelex protocol for DNA extraction (modified from Bouneb et al., 2014). A fragment of the COI gene (covering approximately 670 bp of the 5′ terminus) was amplified using the primers bcdF01 (5′‐TTTTCTACHAAYCAYAAAGATAT‐3′) and bcdR04 (5′‐TATAAACYTCDGGATGNCCAAAAAA‐3′) (Skoracka & Dabert, 2010). If all the mite individuals from one plant were genotyped as MT‐1, we initiated a population of mites collected from the whole plant. If the spike was infested with different *A. tosichella* genotypes, we started separate colonies consisting of mites from single grains, from which all mites were genotyped as MT‐1. There were several cases in which only one female nymph was found on a single grain. In such cases, we established laboratory populations from this single nymph. This was possible because *A. tosichella* reproduces by arrhenotokous parthenogenesis (Miller et al., 2012), where haploid males hatch from unfertilized eggs and diploid females hatch from fertilized eggs. In this way, a single, and even unfertilized, female was able to find a new population by producing haploid sons, picking up their sperm, and then producing diploid female progeny. Subsequently, we genotyped mite individuals again after a few generations of population development. In total, 26 populations from nine different localities were formed. Notably, we did not find MT‐1 mites on barley, triticale, and brome; thus, all populations were initiated using mites collected from wheat. We maintained these populations on wheat plants under constant conditions (22–24°C, 12:12 D/N, 40% relative humidity—RH). We kept the plants infested with mites in rearing cages to prevent contamination among populations. After ~30 days, we again barcoded several randomly chosen individuals from each population to confirm their MT‐1 genotype; no other genotypes were observed in any of the populations. Then, we randomly selected 1000 females from each of the initial populations to establish the stock population, which was maintained under the same conditions as the 26 initial populations (described above) for 4 weeks before they were introduced to our experiments. The plants for all populations and experiments were grown from seeds and cultivated in pots in separate rooms to avoid infestation.

### 2.2 Experimental evolution in constant and fluctuating host environment

We created three host plant selection regimes, each with 10 lines (replicates): constant on *Triticum aestivum* (cT lines), constant on *Hordeum vulgare* (cH lines), and fluctuating by alternation of *Triticum* and *Hordeum* (aTH lines). We established each replicated population with approximately 300 mite individuals sampled from the stock (ancestral) population and transferred them to the potted plants (20 plants per pot). Then, we incubated independent lines within the three regimes in growing chambers under constant conditions, at 27°C, 16:8 D/N, and 60% RH. Every 3 weeks, which corresponds to three MT‐1 generations at 27°C (Karpicka‐Ignatowska et al., 2021), we transferred approximately 300 individuals from each line to a pot with 20 new clean plants according to the selection regime. Then, we measured the response to these three different selection regimes (by assessing mite fitness on different plant species) after the mites evolved for 15, 45, and 60 generations (referred to as generations 15, 45, and 60, respectively, with generation 0 referring to the ancestral population).

### 2.3 Assessing fitness

We measured the fitness of *A. tosichella* MT‐1 from three evolutionary regimes (aTH, cH, and cT) by estimating the population growth rate on four plant species, of which two were involved in the experimental evolution: wheat *T. aestivum* (T) and barley *H. vulgare* (H) (both belonging to the tribe Triticeae). The other two were smooth brome *B. inermis* (B), which belongs to the tribe Bromeae, and rye *Secale cereale* L. (S), which represents Triticeae.

For the experiments, we used wheat, barley, and rye potted plants that were 10–14 days old, and brome plants that were 30 days old, corresponding to approximately the same biomass: leaves at least ca. 100 mm long and 5 mm wide. Owing to the specific growing requirements of *Secale* and *Bromus* plants and technical laboratory constraints, we performed two experimental setups (tests):

Test 1: Estimation of fitness on wheat, barley, and brome of both the stock population (at generation 0) and after the experimental evolution (at generation 45) for aTH, cH, and cT populations. Additionally, we estimated the fitness of generation 15 with respect to aTH, cH, and cT on wheat and barley to determine their responses to experimental evolution changes over time, without testing any specific hypotheses (results presented in Supplementary Material). We transferred 15 females per replicate from either the stock population or each of the regimes (aTH, cH, and cT) to clean wheat, barley, or brome plants (10 plants per pot). Ten replicates of the stock population were tested on either wheat or barley, and 20 replicates were tested on brome. Each evolutionary regime (aTH, cH, and cT) was replicated 30 times for each plant species.

Test 2: Estimation of fitness of aTH, cH, and cT on wheat, barley, and rye at generation 60. We transferred five females per replicate from either stock colony or each of the regimes (aTH, cH, and cT) to clean rye, wheat, or barley plants in specially designed narrow cages containing three plants per pot. Each evolutionary regime was replicated 50 times for each plant species.

We placed the pots (test 1) and cages (test 2) containing the mites in incubators in growing chambers under controlled conditions (27°C, photoperiod 16:8 D/N, 60% RH), and we counted mites after 14 days.

### 2.4 Statistical analyses

All statistical analyses were performed in R version 4.1 (R Foundation for Statistical Computing, 2021) using the “glmmTMB” package to fit the generalized linear mixed models (Brooks et al., 2017).

#### 2.4.1 Population growth rate

The *per capita* population growth rate (*r*) was used as a measure of fitness. This was defined according to the formula: $r=\ln(R_{0})$, where: *R*_0_ is the finite population growth rate, defined as *n*/*n*_0_, wherein *n*_0_ corresponds to the number of females placed on each plant at the beginning of the experiment, and *n* corresponds to the number of mites (being a progeny of *n*_0_ females) counted after 14 days. If *r* > 0, the population size increased, whereas *r* = 0 corresponded to no change in the population size.

Some experimental lines did not survive (*R*_0_ = 0), which prevented the calculation of *per capita* growth rates (*r*) directly by log‐transforming *R*_0_. To tackle this problem, in all statistical models, *R*_0_ was used as a response, which was assumed to originate from a compound Poisson‐Gamma (Tweedie) distribution: $R_{0}∼T_{p}\left(\mu ,\sigma^{2}\right)$, with mean *μ* and variance *σ* ^2^. The mean *μ* was linked to the linear predictor by a natural logarithm function ln(*μ*) = *βX*, where *β* is a vector of model parameters, and *X* is a data matrix. Thus, with this specification, coefficients were expressed on a log scale, which corresponded to *r*.

To test whether the population growth rates differed between different experimental regimes (defined as the host environment: aTH, cH, and cT) and target plant species (T, H, and B) and infer patterns of its change across 45 generations (test 1), a generalized linear mixed model (GLMM) was used with the above‐described error structure, using: (1) a factor coding for the experimental regime (aTH, cH, and cT measured at generation 45) supplemented with one additional level representing the stock population (at generation 0); and (2) the target plant (T, H, and B). To test if the response was consistent across both factors, their interaction was also included in the model. Moreover, to account for any additional unexplained variance due to the variability between the replicates (lines), replicate identifiers were included in the model as random intercepts.

To test whether the population growth rates differed between the experimental regimes on wheat, barley, and rye at generation 60 (test 2), a GLMM was used (with the same error structure as described above), using the experimental regime (aTH, cH, and cT), target plant (T, H, and S), and their interaction as predictors, including replicate identifiers as random intercepts.

#### 2.4.2 Testing predictions

To test our hypotheses, we used the contrasts listed in Table 1. These contrasts represented an effect size (Δ), that is, they are differences in *per capita* population growth rates between specific experimental groups, and thus could be compared directly, despite the different experimental setups. Inferences regarding the contrasts were done by generating population prediction intervals (Bolker, 2008). The procedure consisted of the following steps:

1. The GLMM effects model was reformulated into a cell means model (by fitting individual means for each combination of the factor levels).
2. Parameters were estimated by maximum likelihood.
3. Normal multivariate random sample was drawn from the estimated sampling distribution of a fitted model, using the vector of parameters and the model variance–covariance matrix.
4. Differences (contrasts) between the parameters of interest were calculated.
5. This procedure was repeated 10,000 times to obtain the distribution of contrasts.
6. The t‐statistics and empirical highest posterior density confidence intervals were calculated.
7. The *p*‐values were calculated, considering the direction of the statistical hypothesis (i.e., if the specified contrast’s null reference was above, below, or equal to zero).
8. The *p*‐values obtained in step 7 were adjusted by considering the number of simultaneous comparisons (Benjamini & Yekutieli, 2001).

## 3 RESULTS

### 3.1 Effects of experimental regime and target plant

In test 1, there was no significant effect of the experimental regime (referred to as the host environment: aTH, cH, and cT) (Wald χ^2^ = 3.8, *df* = 3, *p* = 0.2781) on the fitness of the mites. The effects of the target plant and the interaction between the experimental regime and the target plant were highly significant (Wald χ^2^ = 1066.5, *df* = 2, *p* < 0.0001, Wald χ^2^ = 167.0, *df* = 6, *p* < 0.0001, respectively).

Similarly, for test 2, there was no significant effect of the experimental regime (Wald χ^2^ = 1.3, *df* = 2, *p* = 0.5263) on the fitness of the mites, but highly significant effects were observed for both the target plant and the interaction of the experimental regime and the target plant (Wald χ^2^ = 811.8, *df* = 2, *p* < 0.0001, Wald χ^2^ = 361.5, *df* = 4, *p* < 0.0001, respectively).

### 3.2 Generalization and its costs

**FIGURE 1:**
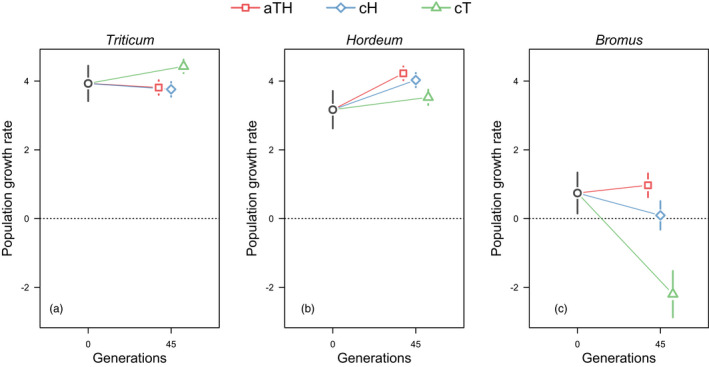
Fitness of *Aceria tosichella* experimental populations evolving in a fluctuating environment (aTH‐lines): alternating wheat (*Triticum*) and barley (*Hordeum*), and in a constant environment: on either barley (cH‐lines) or wheat (cT‐lines), measured as population growth rate on wheat (a), barley (b), and brome (*Bromus*) (c) at generation 45 in relation to the fitness of the stock colony (generation 0) maintained on wheat

As expected, compared to the stock population, the population that evolved in an alternating host environment (aTH) performed significantly better on barley (*Hordeum*) (prediction H1.a: Δ = 1.06, *p* = 0.0013; Figures 1b and 3a). The performance of the mites on wheat (*Triticum*) did not differ from that of the stock population (prediction H1.b: Δ = −0.12, *p* = 1.0000; Figures 1a and 3a).

**FIGURE 2:**
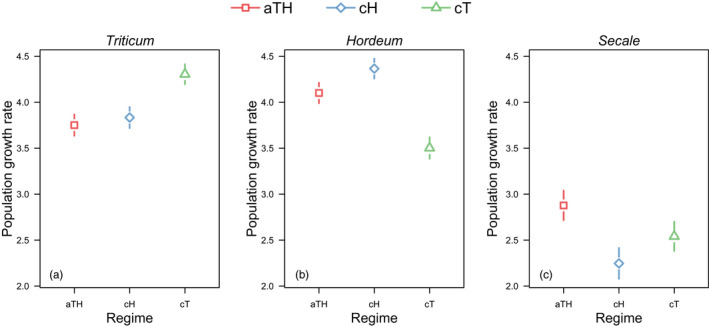
Fitness of *Aceria tosichella* experimental populations evolving in a fluctuating environment (aTH‐lines): alternating wheat (*Triticum*) and barley (*Hordeum*), and in a constant environment: on either barley (cH‐lines) or wheat (cT‐lines), measured as population growth rate on wheat (a), barley (b), and rye (*Secale*) (c) at generation 60

**FIGURE 3:**
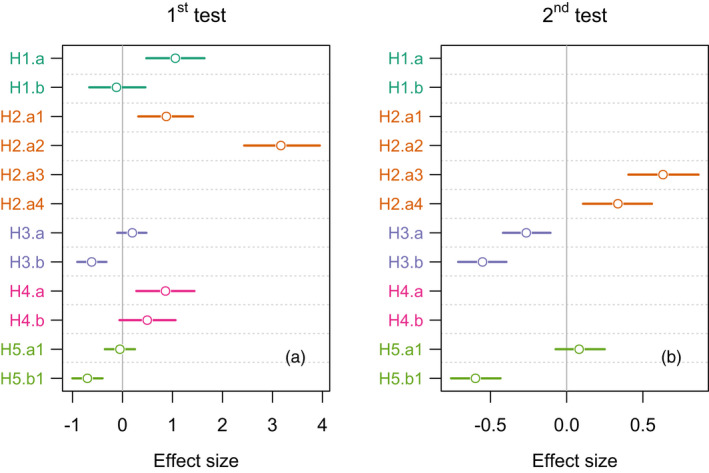
Contrasts reflecting specific predictions after 45 (a) and 60 (b) generations of *Aceria tosichella* evolution. Points represent means, and bars are 95% confidence intervals

Similarly, as predicted in H2.a, the aTH population had significantly higher fitness on the unfamiliar plants: (i) brome (*Bromus*) when compared to the population evolving in the cH (Δ = 0.88, *p* = 0.0049) and cT (Δ = 3.17, *p* < 0.0001) environments; and (ii) rye (*Secale*) (Δ = 0.63, *p* < 0.0001 in cH; Δ = 0.34, *p* = 0.0053 in cT) (Figures 1c and 2c and 3a,b).

In line with the expectation that the generalist will be less fit than the specialist on the shared host, we found that on wheat the aTH population had significantly lower fitness than the cT population, both after 45 and 60 generations (prediction H3.b: Δ = −0.62, *p* = 0.0002, and Δ = −0.55, *p* < 0.0001, respectively; Figures 1a and 2a and 3a,b). However, the aTH population on barley performed no worse than the cH population after 45 generations (prediction H3.a: Δ = 0.20, *p* = 1.0000; Figures 1b and 3a), but after 60 generations, the fitness decreased significantly in comparison to cH (prediction H3.a; Δ = −0.27, *p* = 0.0016, Figures 2b and 3b).

### 3.3 Specialization and its costs

We expected that evolution in constant environments (cH and cT) would increase the fitness of the evolving populations in their environments, compared to the fitness of the ancestral population (stock) in the same environments. In line with this prediction, we found that the cH population performed significantly better on barley than the stock population (prediction H4.a: Δ = 0.86, *p* = 0.0092; Figures 1b and 3a). However, the performance of the cT population on wheat was no better than that of the stock population (prediction H4.b: Δ = 0.50, *p* = 0.1691; Figures 1a and 3a).

We obtained mixed evidence for the expectation that evolution in a constant environment entails the costs of being less fit in environments to which other herbivores are better adapted. In line with this prediction, we observed that, compared to the aTH populations, the performance of the cT population was significantly lower on barley after 45 (prediction H5.b1: Δ = −0.70, *p* < 0.0001; Figures 1b and 3a) and 60 generations (prediction H5.b1: Δ = −0.60, *p* < 0.0001; Figures 2b and 3b). However, after 45 and 60 generations, the fitness of the cH population on wheat was not worse than that of the aTH population (prediction H5.a1: Δ = −0.05, *p* = 1.00; Δ = 0.08, *p* = 1.00, respectively; Figures 1a and 2a and 3a,b).

The differences in the patterns of responses to evolution over time, that is, with respect to the fitness of the populations between the treatments, at generations 15 and 45, are reported in the Supplementary Material (Figure S1).

## 4 DISCUSSION

### 4.1 Host use efficiency in fluctuating and constant conditions

The impact of herbivore pests on agriculture depends on many aspects of their biology and ecology, for example, their abundance, mode of dispersal, interactions with symbiotic microorganisms and natural enemies, ability to vectoring plant pathogens, and overall niche breadth. All these factors influence their ability to efficiently utilize crops (Hoy, 2011; Rusch et al., 2016). In this study, we focused on a wheat pest, wheat curl mite, *Aceria tosichella* (genotype MT‐1), which is a vector of *Wheat streak mosaic virus* that has devastating economic effects in wheat‐growing countries globally (Singh et al., 2018; Tatineni & Hein, 2018). We experimentally evolved the mite population in alternating and constant host environments and demonstrated how these contrasting conditions affect the fitness of this crop pest.

The experiment was initiated from a population of mites, presumably preadapted to wheat, because the stock colony was established from several wild wheat‐associated populations and was maintained on this host in the laboratory before the experimental evolution. Notably, wheat is one of the basic hosts of *A. tosichella* MT‐1 (Navia et al., 2013; Skoracka et al., 2017; Skoracka, Rector, et al., 2018). However, it is crucial to recognize whether the mite has the potential to feed on other plant resources because wheat only serves as a temporary resource for the mites due to seasonal fluctuations. Recent research has shown that other plant species can serve as temporary reservoirs (stepping stones) for the mite (Laska et al., 2021), although no study to date has considered whether and how environmental heterogeneity can affect the mite pest potential.

#### 4.1.1 Fluctuating environment

As expected, at fluctuating environment, consisting of temporally alternating wheat and barley, the mite performance on wheat did not change, but it considerably enhanced its performance on barley, thereby increasing its ability to explore different plant resources. More importantly, the population that evolved in the alternating environment performed significantly better on the plant species not encountered during experimental evolution (i.e., rye and brome) than populations that evolved under constant host conditions. This suggests that when *A. tosichella* MT‐1 experiences a heterogeneous environment in the field, it has the potential to expand its host range by including novel plant species. Further research is needed to elucidate the extent of this expansion and the mechanisms underlying this phenomenon. If generalization is a consequence of selection toward the general stress management associated with various kinds of environments, for example, all‐purpose host defense mechanisms (Hardy et al., 2020), we can expect the invasion potential toward new hosts to be particular. Phylogenetic evidence that generalization enables the colonization of novel hosts (Hardy, 2017; Nylin & Janz, 2009) suggested that potential niche breadth of generalists can indeed be broad. If as a result of generalization, populations harbor adaptations to a set of hosts, we can expect that their genetic variance will enable their rapid adaptation to novel hosts (Barrett & Heil, 2012; Bolnick et al., 2003, 2007; Fox & Morrow, 1981; Loxdale et al., 2011). For example, the generalist two‐spotted spider mite, *Tetranychus urticae* Koch, whose subpopulations perform well only on a subset of potential hosts, in the course of experimental evolution gained an ability to shift rapidly to novel host plants by evolving highly efficient, specific detoxification‐based adaptations, which is a characteristic of specialist herbivores (Salehipourshirazi et al., 2021). Distinguishing which of these two scenarios is more likely for the *A. tosichella* MT‐1 generalist population analyzed in our study would require further experiments combining experimental adaptation and genomic tools.

Irrespective of the underlying mechanisms, the breadth of the niche may be critical in ensuring the pest’s survival and persistence in farmlands when wheat may be temporarily absent due to agricultural practices, such as harvesting or crop rotation. Another possibility for arthropod pests’ persistence could be a dormant stage (diapause) that synchronizes pests with resource availability, and this adaptation has been found in several eriophyoid species (Sabelis & Bruin, 1996). However, this has not yet been confirmed for *A. tosichella*. Therefore, in fluctuating agricultural environments, the subsistence of *A. tosichella* populations most likely depends on their potential to use different plant resources, some of them being sink habitats that function as temporary stepping stones (Laska et al., 2021).

Generalists undoubtedly gain from wide environmental tolerance and their ability to exploit multiple resources. They also have the potential to increase abundance in diverse landscapes (Jonsen & Fahrig, 1997), and are less susceptible to habitat fragmentation, disturbance, and loss than specialists (Büchi & Vuilleumier, 2014; Steffan‐Dewenter & Tscharntke, 2000; Zabel & Tscharntke, 1998). Pest performance under a broad range of conditions can, however, be costly. In our experiment, the enhanced range of exploited plant resources in the population from the alternating environment was associated with certain expenses of being less fit on wheat and barley than the populations that adapted to these hosts, although on barley the costs became evident later, at generation 60. Initial adaptation to barley occurred nearly as efficiently in the alternating environment as when barley was the only host across generations, but the difference became apparent as the specialist continued to evolve after generation 60. It has been predicted (Holt, 1996; Whitlock, 1996) that adaptation to a specific resource should be faster in specialists because a greater proportion of specialists are exposed to selection to that resource. Because the population of *A. tosichella* subjected to alternating environments was less often exposed to selection on barley, selection on this host was expected to be less efficient.

Costs of generalization have been documented for phytophagous insects, bacteria, aphid parasitoids, and solitary bees, and indicate that generalists may be less efficient than specialists in exploiting the resources they have in common (Bernays, 2001; Dykhuizen & Davies, 1980; Straub et al., 2011; Strickler, 1979). However, many other studies have demonstrated opposite results, indicating positive relations between niche breadth and performance (e.gBruns et al., 2021; Futuyma & Philippi, 1987; Huey & Hertz, 1984; Palaima & Spitze, 2004). Thus, the importance of direct fitness costs in maintaining niche breadth remains an unresolved key question (Berenbaum, 1996; Bernays & Graham, 1988; Fry, 1996; Futuyma & Moreno, 1988; García‐Robledo & Horvitz, 2012; Hardy et al., 2020; Visher & Boots, 2020). Nevertheless, regardless of whether or not a generalist pays the costs of lower performance, generalists in heterogeneous agricultural systems should have some advantages over specialists due to their high tolerance and ability to deal with environmental heterogeneity created in farmlands.

#### 4.1.2 Constant environment

In a constant environment, we expected that the fitness of populations that evolved on single hosts would considerably increase in comparison to the ancestral population. This was confirmed for the barley‐adapted population, indicating that the mite has the potential to specialize and increase the efficiency of host use when it encounters monocultures in the field. Compared to the stock population, the wheat‐adapted population also enhanced its fitness, although not significantly, which confirmed that the stock had already been adapted to wheat. Additionally, the wheat‐adapted population attained a significantly higher fitness on wheat compared to the barley‐specialized population and the population evolving in an alternating environment.

*Aceria tosichella* is an important pest in wheat monocultures in most wheat‐growing regions of the world (Navia et al., 2013; Skoracka, Lopes, et al., 2018; Skoracka, Rector, et al., 2018; Skoracka et al., 2014; Tatineni & Hein, 2021). It has been known that agricultural intensification and transformation of landscapes into monocultures increase the risk of pest outbreaks and exacerbate yield losses due to pest damage (Grab et al., 2018; Meehan et al., 2011; Rusch et al., 2016; Tscharntke et al., 2005). Additionally, crop diversity is known to reduce the abundance of pests (Kheirodin et al., 2020), whereas monocultures boost the likelihood of their aggregation and high densities (Dong et al., 2020). Therefore, it is crucial to know whether any crop pest has the potential to change its diet breadth toward specialization or generalization in a specific host environment.

The high performance of specialist species in their optimal habitats is often associated with the expense of their lower performance in other habitats (Futuyma & Moreno, 1988; Levins, 1968; Wilson & Yoshimura, 1994). Likewise, in our study, the wheat‐adapted population paid the cost of lower performance when using barley, as it portrayed significantly lower fitness than the population evolving in the alternating environment. However, the fitness of the barley‐adapted population on wheat did not differ from that of the population adapted to alternating conditions. These asymmetric responses of the barley‐adapted and wheat‐adapted populations resulted from the prior adaptation of the ancestral population to wheat. The population that evolved on barley increased its fitness on barley but did not decrease its fitness on wheat, similar to the population that evolved in the alternating conditions. Thus, considering that both the populations had similar performances on wheat and that the wheat‐adapted population performed well on wheat, our results confirmed that wheat is the main and most preferred host for *A. tosichella* MT‐1. Because the mite is often associated with cereals, it seems obvious that it should be able to use other grass species in the absence of cereals. Indeed, it has long been considered a generalist pest (e.g., Skoracka et al., 2014). However, a recent study indicated that wild grass species (viz. smooth brome) may act only as a sink habitat that functions as a temporal steppingstone allowing for the persistence of a specialist when the source habitat is gone; however, evolutionarily rescue of the pest in such suboptimal habitat is not possible (Laska et al., 2021). The cited recent study and the results presented here strongly indicate that *A. tosichella* MT‐1 is a wheat specialist, rather than a grass generalist.

### 4.2 Responses to experimental evolution over time

It should be emphasized that the observed responses to experimental evolution in a given environment and the associated potential costs of adaptation to environmental conditions change as evolution progresses. This may have affected the interpretation of the results and conclusions. In our experiment, for example, the costs of being a generalist became evident at generation 60; however, the cost was undetected at generation 45. Additionally, in this study, we portrayed how the responses to each environmental regime changed over time, using three time points: generations 0, 15, and 45 (Figure S1). Notably, our results were contradictory to the findings of Magalhães et al. (2009), who revealed that the spider mite *T*. *urticae* adapted to novel host plant species within 15 generations, and no further evolution was observed at generation 25. In our experiment, the responses to different evolutionary regimes did not reach a plateau within a few generations. Such knowledge may have viable practical implications because it can provide a more optimal schedule for further evolutionary experiments of such species, by allowing a sufficient timeframe for adaptation.

### 4.3 Practical agricultural implications

Altogether, our results indicated that the niche breadth of *A. tosichella* MT‐1 evolved in response to the level of heterogeneity of the host environment. In constant host environments, the mite had the potential to evolve toward being a specialist, whereas in the alternating host environment, it acquired the ability to explore different plant species, thus evolving toward a generalist.

Although our experiment focused on a particular crop pest, we believe that they have broader implications and may be applicable to other crop pests associated with similar environments. Notably, our results indicated that homogeneous agricultural conditions intertwined with heterogeneous conditions can sustain pests that have an evolutionary potential to respond quickly to fluctuating conditions by changing their ability to use plant resources. Therefore, agricultural practices may create conditions that influence the evolution of pests’ niches.

The most important implication and conclusion of our study is that environmental variability in agricultural systems may lead to pest generalization, which is expressed as the ability to include many plant species, as well as novel ones, into a pest’s niche. We should be aware that other human activities may also increase the risk of spread of unwanted species that are fit in such agricultural environmental conditions (Karlsson Green et al., 2020; Silva et al., 2020; Yang et al., 2021). First, due to increasing trade, many herbivorous pests are introduced accidentally into new areas and continents. Species that have wide niche breadths may be a potential threat as an invasive species in heterogeneous environments. Second, because of climate change, many crops are introduced to new areas, and native pests with generalist potential may be a potential threat to these new crops. Currently, both scenarios occur in agriculture commonly.

## CONFLICT OF INTEREST

The authors declare no conflict of interest.

## Supporting information

Supplementary Material

## Data Availability

Data for this study are available in Dryad Digital Repository: https://doi.org/10.5061/dryad.q83bk3jkb.
