# Supplementary material for: Effective specialist or jack of all trades? Experimental evolution of a crop pest in fluctuating and stable environments
Source: Evol Appl. 2022 Mar 8;15(10):1639–52. doi: 10.1111/eva.13360 (PMC9624081; doi:10.1111/eva.13360)
Supplement: Supplementary file 1 — Supplementary Material [file EVA-15-1639-s001.pdf]

# Supplementary material

## Effective specialist or jack of all trades? Experimental evolution of a crop pest in fluctuating and stable environments

### Appendix S1 Supplementary results

*The differences in patterns of responses to evolution over time*

We observed some differences in patterns of responses to evolution over time. On wheat, aTH and cH populations showed similar patterns, i.e. at generations 15 and 45 their performance did not differ significantly compared to the stock. The fitness of the cT population at generation 15 was similar to that of the stock, but at generation 45 it was higher than at generation 15 (Fig. 1).

On barley aTH and cH populations again had a similar pattern, i.e. at generation 15 their performance did not differ significantly from performance of the stock, but at generation 45 it was significantly higher, as compared to the stock. In the aTH population, fitness at generation 45 was also significantly higher compared to that at generation 15. The fitness of the cT population did not change during evolution (Fig. 1).

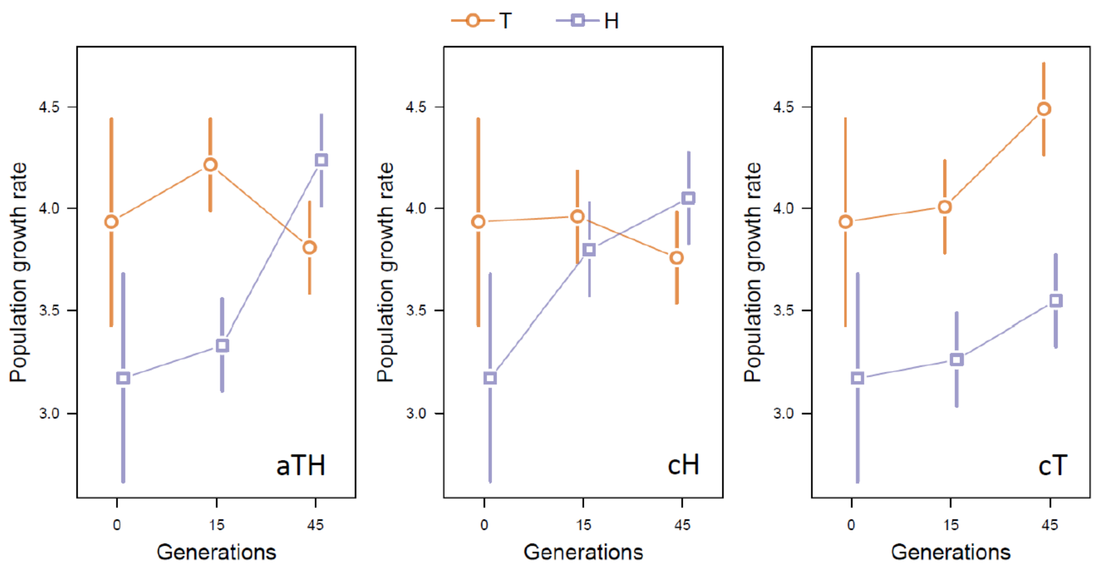


Fig. 4. Fitness of experimental *Aceria tosichella* populations evolving in a fluctuating environment (aTH-lines) on alternating wheat (*Triticum* - T) and barley (*Hordeum* - H), and in a constant environment, either on barley (cH-lines) or wheat (cT-lines), measured as population growth rate on wheat and barley at generations 15 and 45, in relation to fitness of the stock colony (generation 0).
